# An exploration of the attitudes and views of general practitioners on the use of video consultations in a primary healthcare setting: a qualitative pilot study

**DOI:** 10.1017/S1463423618000361

**Published:** 2018-06-18

**Authors:** Ratan S. Randhawa, Joht S. Chandan, Tom Thomas, Surinder Singh

**Affiliations:** 1 Medical Student, Research Department of Primary Care and Population Health, Hampstead, UCL, London, UK; 2 Academic Foundation Year Doctor, City Hospital Birmingham, Birmingham, UK; 3 Foundation Year Doctor, Queen Elizabeth Hospital, Birmingham, UK; 4 Clinical Senior Lecturer/General Practitioner, Research Department of Primary Care and Population Health, Hampstead, UCL, London, UK

**Keywords:** accessibility, primary healthcare, qualitative, technology, video consultation

## Abstract

**Background:**

In 2014, in the United Kingdom, the government made a commitment to spend £3.6 million on the introduction of Skype video calling consultations in general practice, however the efficacy of such technology has not yet been explored fully.

**Aim:**

The study aimed to explore the views and attitudes of General Practitioners (GPs) towards video consultation in primary care; specifically, in three broad areas

- The benefits of video consultations to patients and healthcare professionals.
- Potential problems with video consultation and its implementation.
- The cost-effectiveness of video consultation in this setting.

**Method:**

A convenience sample of the views of 12 general practitioners across two primary care centres in North London were identified using topic guide based semi-structured interviews. A thematic framework approach was used to analyse the data collected to isolate main and sub-themes.

**Findings:**

Three main themes were identified

1. Technology – GPs expressed concerns about the ability of patients to use technology, the availability of technology and the quality of technology available.
2. Utility – encompassing GP’s ideas about the usefulness of video consultations to patients, practitioners and the doctor–patient relationship. GPs presented mixed views on the extent to which video consultation would be useful.
3. Practicality – covering the views of GPs on implementation and effects on workload. GPs unanimously felt that it was not a practical substitute for face-to-face consultation. There were mixed feelings about it being used as an alternative to telephone consultation.

**Conclusion:**

GPs did see potential benefits to using video consultations but also expressed concerns that need to be addressed if they are to have full confidence in the system. The views of those who are going to use video consultation as a means of increasing patient access are paramount if such tools are to be a core part of primary care.

## Introduction

With the advent of better communication modalities in the 21st century, there is growing emphasis on how best clinicians and patients can interact in quick and efficient ways while maintaining safety and overall governance. In particular, the potential for telemedicine to improve accessibility and aid delivery of healthcare to patients has been discussed since the 1990s (McLaren and Ball, 1995; Wootton, 1998). Countries such as Australia, China and Tanzania have piloted video consultation technologies in an effort to improve accessibility to healthcare particularly in rural areas (Hartley, 2012).

Globally, literature suggests that telemedicine reduces barriers to primary care for the general population. In India, Dasgupta and Deb (2008) demonstrated the positive uptake of mobile consultation in the hospital setting. Although limited by sample size, the same results were validated and reproduced in other studies such as Park *et al*. (2004) and Jiwa and Meng (2013). Although telephone consultations are popular around the world, video consultations have now become accepted practice in the healthcare systems of developed nations such as United States and Australia.

Here, in the United Kingdom, the government has committed to incorporating video consultation into the Seven Day GP Access Plan, allocating £3.6 million to run a 230-practice pilot in the United Kingdom (Lind, 2014).

Video consultations were first used as a method of communication between healthcare professionals who could not consult face-to-face due to constraints such as time, location and availability (Dasgupta and Deb, 2008). In 2004, a single randomized blinded prospective trial (Meyer *et al*., 2008) compared the efficacy of video-based communication versus telephone-only consultations for decision making on an acute stroke unit in the United States. The results suggested doctors employing video consultation made a greater number of correct treatment decisions than those that employed telephone-only consultations (98 versus 82%) (Capampangan *et al*., 2009). This suggests that video consultation used in the correct circumstances could potentially be a more effective consulting tool than mobile consultations used in current practice. Similar results were found in Germany within an acute healthcare setting (Handschu *et al*., 2008).

Although extensive research has been undertaken into the efficacy of these modalities in the clinical setting, implementation and uptake of telemedicine is dependent upon the attitudes of the key stakeholders. Current literature does not fully explore the attitudes or perception of the healthcare professional to this technology, about which there is a dearth of information.

A literature review conducted at the time of the study of the current published literature on the topic of video consultation in the PubMed/MEDLINE and the Cochrane Library databases yielded 1239 studies. However following a stringent inclusion criteria, using appropriate search terms (Appendix 1), only one study explored the views and attitudes of general practitioners on the use of video consultations in a primary healthcare setting (Jiwa and Meng, 2013).

Jiwa and Meng (2013) examined the attitudes of Australian general practitioners towards video consultations. In total, 102 general practitioners were invited to view six different video vignettes with patients who had both acute and chronic conditions. Respondents were asked their views on the process of virtual consultations – including its perceived value. The study concluded that all the GPs were comfortable with video consultations. GPs working in bigger practices favoured video consultations more and the older, more experienced GPs less so. However, there were some important limitations which the authors acknowledged; the video vignettes were non-interactive and thus could not truly represent the dynamic nature of a consultation.

Considering the existing limitations in previous literature and increased funding supporting video consultations in the United Kingdom, a study exploring the views of UK-based general practitioners working within the NHS will offer a new perspective in regard to opinions and views of health professionals on video consultation.

## Method

### Sample/setting

In total, 12 semi-structured interviews lasting 45–60 min with different GPs were conducted using a pilot tested topic guide (see Appendix 2). The interviews took place across two North London GP practices in May 2014. A convenience sample of GPs was taken and potential participants were identified and recruited via email/face-to-face through a single informant GP at one of the practices using a ‘snowballing’ method. All approached GPs took part in the study.

### Ethics/relationship between researcher and participants

Ethics approval was received from the University College London (UCL) ethics board in January 2014 (approval number: 5314/001).

Before field research could take place, approval had to be given by the specific office of NHS Trust Research and Development (R&D) for the area in which the study was going to be based and from which participants were to be recruited. Interviews were conducted in private rooms and the transcripts were anonymised. Both verbal and written informed consent was obtained from the participants before interview. Participants were reassured that their transcripts – and any quotes which appeared in the final report – would be anonymised.

The study was registered with the University College London Data Protection Officer and is still bound by the Data Protection Act 1998. The primary researcher, R.S.R., who is a male medical student trained in qualitative methods during his intercalated B.Sc. S.S., the primary supervisor supporting R.S.R., has an extensive history in published qualitative work. Before study commencement, R.S.R. had developed a relationship with a small number of the GPs interviewed, whom he had shadowed as a medical student. The work was done primarily as means of a thesis for a B.Sc., and this was relayed to the participants.

### Data collection

The data collected originated from the semi-structured interviews conducted in the GP practices. The 12 interviews were conducted across the two GP sites by RR alone after verbal and written consent. The topic guide consisted of collecting demographic data pertaining to age, gender, ethnicity and years working as a GP. Then the interviews consisted of open questions exploring the study aims with more in-depth questioning in the areas of interest brought up by the participants. The term video consultation was deliberately undefined to maximise breadth of participants’ responses relating to their interpretation and use of this type of consultation. Interviews ranged from 45 to 60 min in length and were recorded.

### Analysis

Interviews were transcribed verbatim, and data analysis was supported by the use of NVIVO (v10). Data analysis was conducted using a thematic framework approach (Pope *et al*., 2000) to allow for analysis of themes and trends discussed by the participants. The data were analysed repeatedly using methods such as constant comparison, and subsequent interviews benefited from subtle iterations of the topic guide where emerging themes were identified. The framework approach developed by Braun and Clarke (2006) was adapted for use in this analysis:

1. Familiarisation with the data – R.S.R. transcribed the interviews on the day of the interviews to minimise researcher recall bias. The transcripts were re-read before analysis.
2. Creating primary codes for the data – R.S.R. and S.S. identified possible themes after re-reading of the transcripts.
3. Exploring the data for themes – R.S.R. then coded and indexed the data by common themes.
4. Reviewing the identified themes – the themes were then reviewed by S.S. and final set of agreed themes were compiled.
5. Creating a final report – the final report was written by R.S.R., J.S.C., T.T. and S.S.

Member checking took place as following the analysis; participants were sent a copy of the transcript and the researcher’s interpretation. Participants were asked if they wished to make any changes, as all 12 GPs responded they would like no changes to be made.

## Findings

The participants’ demographic details are described in Table 1 (not all included to maintain anonymity). Three main themes were identified with their respective sub-themes (Table 2).

**Table 1:** Demographic data

Interview number	Sex	Age (years)	Years GP experience
1	Female	48	15
2	Female	38	5
3	Female	62	30
4	Male	39	9
5	Male	45	10
6	Male	46	15
7	Female	54	19
8	Male	47	12
9	Male	31	2
10	Male	50	18
11	Female	30	1
12	Female	36	6

**Table 2:** Themes and sub-themes

Theme	Sub-theme
Utility	Access
	Patient applicability
	Examination
	Doctor–patient satisfaction
Practicality	Time
	Video consultation versus face-to-face consultation
	Confidentiality and security
	Inevitability
	Cost
Technology	Technological literacy
	Availability of technology
	Quality of technology

### Theme 1: utility

All the participants felt that video consultation could be useful to patients.

*‘I haven’t really seen it in practice but I’m sure there are number of potential benefits of using it.’*

((5) Male, 45 years old)

#### Access

Some participants felt the use stemmed from improved access to GPs for patients.

*‘It’s easy, it’s access, it’s you know it’s easy for patients, some patients do come to the surgery and they want video consultation… the general complaint right now is “I can’t see my GP.” No access, no access, no access’.*

((1) Female, 48 years old)

For some participants this improved access was reciprocal as it saved GPs time to see their patients.

*‘It not only improves access for patients but also for doctors. I mean it’d be much easier for me to go to a patient’s home via the computer than to physically drive there’.*

((5) Male, 45 years old)

Another GP explored the option that other members in the multidisciplinary should have access to the video services such as translators as it could improve the patient experience. This explored the concept that video consultation may be more useful for specific patient cohorts.

*‘I’ll tell you what though the translators idea is quite good. Because then the translators can work from home and be accessed by multiple practices you know’”*

((9) Male, 31 years old)

#### Patient applicability

Participants had mixed feelings regarding which patients would benefit most from video consultation.

*‘I don’t think it would help in all cases…Management of ongoing problems where you need to speak to the patient, bit of visual assessment would be good as well. But difficult to manage patients who have mental health problems because I think, face to face consultation is a bit different from video with mental health’.*

((1) Female, 48 years old)

In contrast to the above quote, another participant believed that it would help mental health patients.

*‘Definitely for follow up consultations, even with some mental patients or patients with depression. They don’t really have any physical problems and just need a small assessment’.*

((5) Male, 45 years old)

In addition, there was a belief that patient knowledge and competence was an important factor for successful video consulting.

*‘…the other thing is fine but the patient on the other hand has to be competent enough to understand what you are telling him, there could be an educated person, if they’re not then it’s difficult’.*

((1) Female, 48 years old)

Many of the participants believed that the housebound would benefit most from video consultation:

*‘The positive I suppose is for the patient that’s housebound’.*

((3) Female, 62 years old)

*‘It’s aimed at patients who can’t make it to the surgery so I suppose patients who can’t get out of the house, housebound patients’.*

((4) Male, 39 years old)

#### Examination

Clearly an examination is not possible using video-technology, however visualising the patient and their condition can increase confidence in the general practitioner’s mind regarding diagnosis:

*‘It would help to see the patient sometimes because sometimes you know, there’s a level of uncertainty (hmm)…But yeah perhaps if I could’ve seen it then I could’ve told them it was nothing to worry about rather than have to call them in (yeah) so video could be helpful there’*

((6) Male, 46 years old)

*‘I mean it’s only really going to be useful for rashes and things like that otherwise you need to see the patient…It’ll be useful for the odd rash or something’.*

((2) Female, 38 years old)

Many participants expressed concern that the lack of physical examination was a drawback to video consultation and left the assessment of the patient somewhat incomplete:

*‘Well not being able to physically assess the patient is definitely a problem. I mean even if it’s a rash… you could diagnose occasionally but video consultation won’t show you all the characteristics, whether the skin is raised or rough’*

((10) Male, 50 years old)

Interestingly due to these limitations many of the GPs felt that they would not be comfortable referring patients from a video consultation directly to secondary care. With a GP feeling it could create a barrier to face-to-face consultations:

*‘I don’t think I’d refer to secondary care because you can’t really tell for sure unless you do a face-to-face, only for a small range of conditions you know. It might even create a barrier to primary care because like telephone consultations you end up telling them to come in for a consultation’.*

((11) Female, 30 years old)

#### Doctor–patient satisfaction

Many participants felt that the doctor–patient relationship would be affected by using video consultation when compared with face-to-face consultations, which may affect the patient and doctor’s satisfaction:

*‘When you see a patient face to face you can negotiate and you can plan with the patient that look “I don’t know what’s happening but I think there is something going on, we’ll keep an eye on you, come see me in two weeks” that’s more satisfying for the patient than Skype’.*

((1) Female, 48 years old)

Whereas others felt that a strength of the video method was the ability to see your doctor which could be an improvement on current telephone methods for patient satisfaction.

*‘I suppose just being able to see your doctor could go a long way to improving the doctor-patient relationship’.*

((4) Male, 39 years old)

### Theme 2: practicality

Practicality refers to the attitudes and perceptions of GPs to the real world application of video consultations.

#### Time

Many GPs thought that the introduction of video consultation would not save any time in general practice and could ultimately waste time if you ultimately have to bring the patient in.

*‘I find that even with telephone consultations that are supposed to be shorter often they’re not because you’re not seeing things and the patient still wants reassurance and you also want the reassurance that you’ve done a full assessment…if it is a proper complaint then it doesn’t really save any time it’s just that convenience thing’.*

((12) Female, 36 years old)

Interestingly some GPs felt that as a result this could ultimately lead to a greater workload for GPs and nurses to get their observations recorded and tasks relating to reaching quality outcome framework (QOF) targets ultimately taking up more time.

*‘I suppose we’d end up having to book even more patients in with the nurse for their check-ups because usually we do them (blood pressure checks) during consultations. But yes if Skype consultations reduce the amount of people coming in then I suppose it would be harder to reach our QOF targets and it might make the nurses day busier…I don’t think they’d reduce the workload…I mean it might increase the GPs workload’.*

((4) Male, 39 years old)

Other participants thought that there could be some time-saving aspects to video consultation relating to travel time.

*‘I mean it’d be much easier for me to go to a patient’s home via the computer than to physically drive there …I think it could help reduce home visits to those that are necessary and help reduce the face-to-face consultations to the more necessary ones too’.*

((5) Male, 45 years old)

#### Video consultation versus face-to-face consultations

Many of the participants expressed views that the video consultation was not an appropriate replacement or alternative to face-to-face consultations due to barriers with examination and patients being discouraged to see their GP.

*‘Yes I don’t think there’s anything that can replace the true consultation in person to be perfectly honest?’*

((6) Male, 46 years old)

Interestingly a few participants thought that video consultation could hinder the learning experience that some GPs gained from face-to-face consultations.

*‘The lack of clinical experience will most probably hinder the learning …it will prevent younger general practitioners from picking up those patterns to recognise in patients’.*

((9) Male, 31 years old)

However, in comparison with telephone consultations, participants felt the visual aspect of the video consultation would increase certainty in their assessment of patients.

*‘perhaps if I could’ve seen it then I could’ve told them it was nothing to worry about rather than have to call them in’.*

((6) Male, 46 years old)

Some participants felt that it may improve rapport and confidentiality as on camera you can clearly identify who you are speaking to.

*‘I mean on a telephone you can’t even tell if you’re really talking to the correct person. There’ve been times when the son of a patient will answer the phone and they just sound so alike you can’t tell’.*

((7) Female, 54 years old)

#### Confidentiality and security

Although you can identify who you are speaking to, many of the participants believed that one of the drawbacks of video consultation was that there could be issues with protecting confidentiality as you cannot see the whole room with the webcam.

*‘…especially with a video screen you can’t really tell who’s in the room beyond the boundaries of the (camera angle), whereas with a face-to-face consultation I can control who’s in the room and that will change based on the nature of the problem’.*

((4) Male, 39 years old)

The security of the software also raised concerns relating to the confidentiality of the consultation:

*‘Can’t people hack into webcams? They’d be able to watch the GP or the patient’.*

((7) Female, 54 years old)

#### Inevitability

Even with the barriers to use, many of the participants felt that the implementation of video consulting was inevitable:

*‘This is the way forward, we can’t, we can’t run away from this. It’s happening and it’ll be something we’ll be doing’.*

((1) Female, 48 years old)

#### Cost

Many participants believed that from a cost perspective, the initial cost would outweigh the long-term running costs, but that overall video consultation could prove to be cost-effective.

*‘Well then it (cost) must be quite low because the system itself is free. But then there’s so much to consider, you’ve gotta pay the people to install the system on the system, and then there are the costs of training GPs to use the system’.*

((4) Male, 39 years old)

Whereas others felt that the introduction of video consultation would not save money and that cost-saving was not the major reason to implement it.

*‘…it probably won’t save the NHS any money… but I don’t think it’ll cost the NHS too much in the grand scheme of things either, so I suppose it’s not really about cost, it’s more about trying to improve patient care, and access’.*

((5) Male, 45 years old)

### Theme 3: technology

The third theme related to the technology itself, which the participants felt could create difficulties for the patients using the service.

#### Technological literacy

One obvious limitation of the service related to challenges with technology. There was a belief amongst some participants that the older generation and housebound would not be able to use the technology required to make video consultation effective, however these are the very groups who have, potentially at least, most to gain.

*‘I said the people who need it most are probably the housebound who can’t get to us. Will they be able to use it?’*

((2) Female, 38 years old)

#### Availability of technology

Some GPs also expressed concern about patients having access to the technology required for video consultation:

*‘People have access to a smartphone, or an IPad or something yeah. So I suppose that’ll be one drawback so the elderly are often the people that do ask for telephone consultations and things because they can’t make it to the surgery but I’m not sure that they would have access to that’.*

((12) Female, 36 years old)

#### Quality of technology

Many participants believed that the quality of the image was an important factor which should be considered when determining whether video consultation could be considered useful:

*‘I’m not sure how good it would be but if you put it right up to the thing (yeah), to the whatever it is they do, depends on the image quality but you could do that’.*

((3) Female, 62 years old)

Other GPs voiced concerns that video consultations would be open to a number of technical issues, primarily issues with connectivity:

*‘Ok yeah I mean it could work, but Skype has a lot of problems. Connectivity issues, sound and image’.*

((8) Male, 47 years old)

### Summary of the key findings

There were three main themes:

- Utility – all participants felt that video consultation could be useful to their patients. Although it could improve between patients and GPs, it may be more appropriate for certain patient groups over others, and could lead to barriers relating to the ability to examine patients affecting the doctor–patient relationship.
- Practicality – participants had mixed views about the potential for time-saving benefits. GPs felt it was not a substitute to face-to-face consultations. However, it may be superior to telephone consultations.
- Technology – participants felt that the success of the service ultimately depends on the literacy, availability and quality of technology accessible to the patients who need the service most. Participants felt that the ultimate success of the service is dependent on the availability and quality of the technology as well as the literacy of the patient with regards to these technology-driven consultations.

## Discussion

This was a pilot study designed to explore the views of general practitioners about video consultations, with a particular emphasis on the acceptability of the technology. This is an area where there is a dearth of information – especially with regard to *how* GPs perceive this growing, inevitable development of video consulting.

The results of Jiwa and Meng’s (2013) study assessing GPs views on video-consultation concluded that the majority of the GPs were comfortable with video consultation, but that some had expressed reservations, which were then unexplored. Similarly, in our study GPs saw the potential for benefit; however some of these health professionals had significant doubts such as increase in workload.

Capampangan *et al.’s* (2009) single-blinded randomised control trial demonstrated video consultation used in an emergency neurology/stroke unit can be more effective than telephone consultation from a diagnostic perspective. Once again whether this can be translated into similar benefits in primary care has yet to be studied. In our study general practitioners certainly thought that there is great potential in the use of video consultations and that for some patients it might improve accessibility for patients.

### Strengths and limitations of the study

The use of convenience sampling was appropriate for the study considering the time constraints of having to carry out the interviews in the study timeframe; however a convenience sample may therefore not be representative of the population studied. Although time efficient in difficult to access groups such as GPs who are busy, the snowball method of recruitment is limited as results may be affected by the relationship between informant GP and the interviewees introducing a sampling bias. Another limitation was that the interviews were conducted during working hours. This may have potentially led to the participants feeling rushed as they had to attend to their own practice work. To alleviate this concern, the interviews were conducted at the practice at a time of their choosing. Furthermore, the small sample size increases the risk of bias in the results. However, this study aimed to be a pilot study to identify areas where further work could be explored.

During the interview and analysis phase, no new themes emerged after 9 of the 12 interviews. It is difficult to say whether interviewing more GPs at different practices would have led to identifying new themes. However, there was also a secondary time constraint on the research, which also meant it would be unfeasible to conduct any further interviews. It is important to note that there is a possibility that new themes may have merged if further interviews were conducted. The member checking process was extremely successful with all interviewees responding and no subsequent changes being made to the transcripts.

The 32-point consolidated criteria for reporting qualitative studies (Tong *et al*., 2007) was considered but not strictly adhered to throughout study design and reporting which may have limited the reliability of data collected. In future work, we would advise strict adherence to similar guidance to ensure reliability of data collection.

There is a possibility that the relationship between R.S.R. and the participants may have affected the data collected. However, being a medical student undertaking an intercalated B.Sc. in primary healthcare R.S.R. shared some understanding of the potential issues the GPs were facing and in turn improve the exploration of the themes posed.

### Application of findings

The results of the study should be considered in the light of the limitations. Although this study’s aim was to explore GPs views, the results do indicate several areas that could be considered when implementing video consultation into practice.

From this study, the barriers to full implementation include:

- Confidentiality issues
- How to integrate video consultation into practice (should video consultation be regarded as similar to telephone consultations or triage?)
- Effect on GP time and workload.
- How will GPs use this modality and the potential costs to the practice.

The results of the study could also be useful to patients who want to know if they should use video consultation for their query. For example, the findings show that some GPs felt that the use of video would not be useful in diagnosing conditions beyond a rash. Patients should be able to look at this research and make a more informed choice as to whether using video consultation is appropriate for their problem and circumstances that they face.

Although this research was undertaken using a small number of respondents in two locations, this study can provide the basis for future work to take place exploring GPs views through quantitative methods.

## Conclusion

The views of general practitioners in the study can be split into three broad themes related to video consultation:

- Utility
- Practicality
- Technology

These themes need to be considered by those thinking of implementing video consultation and considering conducting future research. The themes encompass concerns that GPs had towards video consultation and measures should be taken to reassure GPs or address concerns that they may have. The warnings of GPs about video consultations should also be heeded in future decision making on the subject.

## Financial Support

This research received no specific grant from any funding agency, commercial or not-for-profit sectors.

## Conflicts of Interest

None.

## Appendix 1: Search strategy used for the literature review

Search terms used:

1. ‘Video Consultation’
2. ‘General Practice’
3. ‘Telecommunication’

Inclusion criteria:

1. Studies that mentioned video consultation in some capacity.
2. Studies that focused on the use of video consultation in a primary care setting.
3. Studies that made reference to the opinions of general practitioners on the application of video consultation as an alternative or additional consultation tool.
4. Limited to the last 10 years.

## Appendix 2: Topic guide

Topic guide for interviews

1. Generally, what do you think of video consultation as a tool?
2. What do you think the affect would be on workload?
3. Do you think that it’s a good alternative to telephone consultations?
4. Do you think video consultation will be cost-effective?
5. Do you have any questions for me?
6. Would you like the summary of the findings of the research?

Where the topics implied by the questions are already covered in free-flowing conversation before the question is asked, it is not necessary to stick to a strict regimen of questions.
